# Wild rice harbors more root endophytic fungi than cultivated rice in the F1 offspring after crossbreeding

**DOI:** 10.1186/s12864-021-07587-1

**Published:** 2021-04-17

**Authors:** Lei Tian, Enze Wang, Xiaolong Lin, Li Ji, Jingjing Chang, Hongping Chen, Jilin Wang, Dazhou Chen, Lam-Son Phan Tran, Chunjie Tian

**Affiliations:** 1 Northeast Institute of Geography and Agroecology, Chinese Academy of Sciences, Changchun, 130102 Jilin China; 2 University of Chinese Academy of Sciences, Beijing, 100049 China; 3 Rice Research Institute, Jiangxi Academy of Agricultural Sciences, Nanchang, 330200 China; 4 Institute of Research and Development, Duy Tan University, Da Nang, 550000 Vietnam; 5 Institute of Genomics for Crop Abiotic Stress Tolerance, Department of Plant and Soil Science, Texas Tech University, Lubbock, TX 79409 USA; 6 Key Laboratory of Straw Biology and Utilization of the Ministry of Education, Jilin Agricultural University, Changchun, 130118 Jilin Province China

**Keywords:** Cultivated rice, Microbiota, Endophytic, Wild rice

## Abstract

**Background:**

Rice, which serves as a staple food for more than half of the world’s population, is grown worldwide. The hybridization of wild and cultivated rice has enabled the incorporation of resistance to varying environmental conditions. Endophytic microbiota are known to be transferred with their host plants. Although some studies have reported on the endophytic microbiota of wild and cultivated rice, the inheritance from wild and cultivated rice accessions in next generations, in terms of endophytic microbiota, has not been examined.

**Results:**

In the present study, the endophytic microbial community structures of Asian and African wild and cultivated rice species were compared with those of their F1 offspring. High-throughput sequencing data of bacterial 16S rDNA and fungal internal transcribed spacer regions were used to classify the endophytic microbiota of collected samples of rice. Results indicated that when either African or Asian wild rice species were crossed with cultivated rice accessions, the first generation harbored a greater number of root endophytic fungi than the cultivated parent used to make the crosses. Network analysis of the bacterial and fungal operational taxonomic units revealed that Asian and African wild rice species clustered together and exhibited a greater number of significant correlations between fungal taxa than cultivated rice. The core bacterial genus *Acidovorax* and the core fungal order Pleosporales, and genera *Myrothecium* and *Bullera* connected African and Asian wild rice accessions together, and both the wild rice accessions with their F1 offspring. On the other hand, the core bacterial genus *Bradyrhizobium* and the core fungal genera *Dendroclathra* linked the African and Asian cultivated rice accessions together.

**Conclusions:**

This study has theoretical significance for understanding the effect of breeding on the inheritance of endophytic microbiota of rice and identifying beneficial endophytic bacteria and fungi among wild and cultivated rice species, and their F1 offspring.

**Supplementary Information:**

The online version contains supplementary material available at 10.1186/s12864-021-07587-1.

## Background

Endophytic microbes inhabit plant tissues without causing any obvious damage to their host, and play a crucial role in plant growth, development, fitness and protection [1–3]. These endophytic microbes, including bacteria and fungi, spend a portion of their life cycle inside plants where they normally reside in intercellular spaces, and obtain carbohydrates, amino acids and inorganic nutrients from their host [4]. Despite their beneficial effects on plant growth and development, root-borne endophytic microbes have still been largely unexplored in rice. High-throughput technologies, such as next-generation sequencing (NGS), along with universal primer sets, have greatly facilitated the opportunities to study and characterize endophytic microbiota. Consequently, the sequences obtained from large numbers of microbial taxa have encouraged in-depth analyses of microbial communities to be conducted in taxonomic, phylogenetic and evolutionary studies [5–7]. The microbiota comprising a microbiome can respond and adapt to environmental conditions [8–10]. Microbiota maintain a strong relationship with their host plants, and can affect host metabolism, which may have either a beneficial or detrimental effect on the plant host [11–13]. Cultivation of a single crop variety may have a detrimental impact over time, including increased susceptibility to pathogens [14–16]. In contrast, hybridization has been reported to promote plant stress resistance [17, 18]. Studies have shown that the beneficial physiological traits of hybridized plants are stronger than their original parents [17, 18]. No studies have determined, however, the impact of hybridization on the endophytic microbiomes of roots of plants.

Rice (*Oryza sativa*) is the main food staple for approximately half of the world’s population. Thus, breeding for yield improvement to feed an ever-increasing world population is a critical goal of the rice research community [19–24]. Common wild rice (*O. rufipogon*), a relative of cultivated rice, possesses several unique attributes, including disease and lodging resistance, as well as drought tolerance. Adverse environmental conditions can negatively impact rice growth and development, and consequently affect rice yields worldwide [25–27]. Wild rice is a genetic resource with several advantageous traits that are useful to rice breeders, and can be utilized for trait improvement in cultivated rice varieties [21, 28, 29]. Several recent studies have shown that the endophytic microbial community associated with wild plant species may play an important role in disease resistance [3, 29, 30]. Rice endophytic bacteria may also promote rice growth [10, 31–33]. In this regard, Sun et al. revealed the diversity of the endophytic bacterial community in rice by 16S rDNA sequence analysis using first-generation sequencing technology [31]. Feng et al. reported that the rice endophyte *Pantoea agglomerans* YS19 promoted the growth of rice plants and affected the allocation of host photosynthates [32]. Hybridization of cultivated plant species with wild species can not only transfer beneficial traits from both parents to the next generation but also induce beneficial changes in the composition of the endophytic microbial community present in their offspring, potentially leading to higher yields even under adverse environmental conditions [34, 35].

It is not yet clear, however, how the diversity of the endophytic microbial community changes following the hybridization of wild and cultivated rice species. Therefore, a comparative analysis was conducted to compare the compositions and diversities of the endophytic root microbiomes of cultivated and wild rice species, as well as their hybridized F1 offspring. This analysis will provide a greater understanding of how the endophytic root microbiomes acclimate and respond to crossbreeding and ultimately provide an approach for restructuring the endophytic microbial community to support sustainable agricultural systems for rice production, as well as other crops.

Cultivated rice comprises two main species, Asian cultivated rice (*O. sativa *subsp. *indica* and *O. sativa *subsp. *japonica*) and African cultivated rice (*O. glaberrima*). The Asian cultivated rice varieties originated from nivara wild rice (*O. nivara*) or common wild rice (*O. rufipogon*), while African cultivated rice originated from African wild rice (*O. barthii*). Plant resources were successfully obtained for Asian cultivated rice, African cultivated rice, nivara wild rice, African wild rice, and their hybrid seeds, for use in this study. Specifically, these materials were utilized to characterize endophytic microbial diversities and community structures in Asian and African cultivated and wild rice, and F1 offspring resulted from the hybridization of wild and cultivated species. It would be then interesting to investigate how such crossing of various wild and cultivated rice accessions would allow their F1 offspring to inherit the endophytic microbiota from their parents.

## Results

### Analysis of the Illumina sequencing data

Twelve sample groups were sequenced with each group comprising 4 biological replicates (Additional file 1: Table S1). A total of 1,900,512 paired-end reads of bacterial sequences were obtained after filtering low-quality and other unsuitable sequences. The average number of clean reads was 39,594 per sampled group, with a minimum read number of 30,750. The average length of the reads was 430 bp. The rarefaction curves, displaying the relationship between the number of reads and operational taxonomic units (OTUs) in each sample, exhibited a stable plateau, indicating that the read depth and OTUs were sufficient for further analyses (Additional file 2: Figure S1).

A total of 1,905,330 clean fungal sequences were obtained after the requisite filtering of unqualified reads. The average read number for each sequenced sample was 39,694, with the lowest read number of 30,748 obtained for only one sample (Additional file 1: Table S1). The rarefaction curves again indicated that the sequencing depth in relationship with the number of OTUs was sufficient to continue with further analyses (Additional file 2: Figure S1).

### Taxonomic analysis

**Fig. 1:**
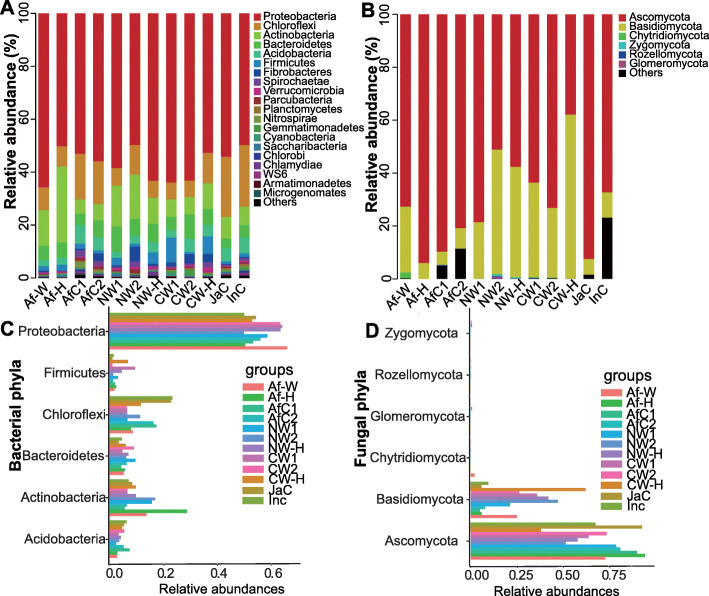
Taxonomy of endophytic bacteria (**a**, **c**) and fungi (**b**, **d**) at the phylum level. Af-W, African wild rice; Af-H, F1 generation of a cross between African wild rice (Af-W) and African cultivated rice (AfC1); AfC1, African cultivated rice No. 2; AfC2, African cultivated rice No. 4; NW1, nivara wild rice No. 1; NW2, nivara wild rice No. 2; NW-H, F1 generation of a cross between nivara wild rice (NW1) and Asian cultivated rice (indica, InC); CW1, common wild rice No. 1; CW2, common wild rice No. 2; CW-H, F1 generation of a cross between common wild rice (CW1) and Asian cultivated rice (japonica, JaC); InC, Asian cultivated rice (Jiangxi indica); JaC, Asian cultivated rice (Jiangxi japonica). Each group had 4 replicates (*n* = 4)

The bacterial and fungal communities were first characterized at the phylum level. The main bacterial phyla in the samples were Proteobacteria, Chloroflexi, Actinobacteria, Bacteroidetes, Acidobacteria and Firmicutes, followed by Fibrobacteres and Verrucomicrobia (Fig. 1a), while the dominant fungal phyla were Ascomycota, Basidiomycota, Chytridiomycota and Zygomycota (Fig. 1b). A comparison of the relative abundance of bacteria revealed that the relative abundance of Chloroflexi was significantly higher in both African and Asian cultivated rice species, relative to the respective wild rice species from which they had originated (Fig. 1c). A similar comparison of fungal phyla revealed that the relative abundance of Basidiomycota was significantly higher in both African and Asian wild rice and their corresponding F1 generations than in their related cultivated rice species (Fig. 1d). Although Glomeromycota had a low relative abundance relative to other phyla, its relative abundance was significantly higher in common wild rice *O. rufipogon* than in all of the other species and hybrids (Fig. 1d).

### Alpha diversity analyses of the endophytic microbial communities of roots in cultivated and wild rice species and their F1 offspring

**Fig. 2:**
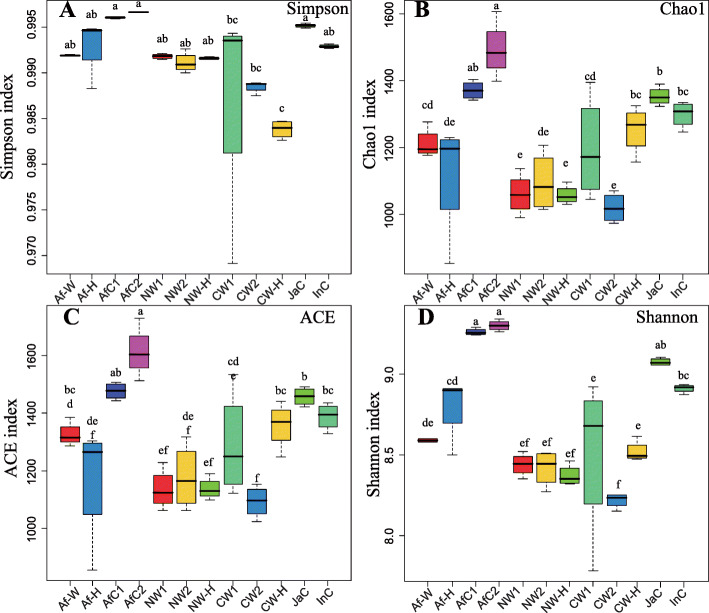
Box plots of the alpha diversity indices of the endophytic bacterial communities of cultivated and wild rice plants, and their F1 offspring. Simpson (**a**), Chao1 (**b**), ACE (**c**) and Shannon (**d**) indices. Each boxplot represents the distribution of diversity present in four replicates (*n* = 4). Af-W, African wild rice; Af-H, F1 generation of a cross between African wild rice (Af-W) and African cultivated rice (AfC1); AfC1, African cultivated rice No. 2; AfC2, African cultivated rice No. 4; NW1, nivara wild rice No. 1; NW2, nivara wild rice No. 2; NW-H, F1 generation of a cross between nivara wild rice (NW1) and Asian cultivated rice (indica, InC); CW1, common wild rice No. 1; CW2, common wild rice No. 2; CW-H, F1 generation of a cross between common wild rice (CW1) and Asian cultivated rice (japonica, JaC); InC, Asian cultivated rice (Jiangxi indica); JaC, Asian cultivated rice (Jiangxi japonica)

Alpha diversity analyses, including Simpson, Chao1, ACE, and Shannon indices, were conducted characterize differences in bacterial and fungal community abundances and diversities in wild and cultivated rice species and their F1 hybrids. Results revealed that alpha diversity indices of the bacterial and fungal endophytic root microbiome of rice were not static but varied among the cultivated and wild rice species and their F1 offspring. As shown in Fig. 2, significantly higher Chao1 and Shannon indices were observed in the compositions of the bacterial communities of both African and Asian cultivated rice species, relative to the same indices measured in wild rice relative and respective F1 offspring (Fig. 2b, d). In contrast, no significant differences in the Simpson and ACE indices of the bacterial communities were observed in both African and Asian cultivated rice species, relative to their related wild rice species and respective F1 offspring (Fig. 2a, c). The bacterial Chao1 and Shannon indices of the African cultivated rice species (AfC1 and AfC2) were significantly higher than the same indices obtained in African wild rice (Af-W) and their F1 offspring (Af-H), while no significant differences in these indices were observed between African wild rice (Af-W) and the F1 offspring (Af-H). Similarly, the Chao1, ACE and Shannon indices for bacterial taxa in Asian cultivated rice accessions (JaC and InC) were significantly higher than in nivara wild rice accessions (NW1 and NW2) and their F1 offspring (NW-H), while no significant difference was observed between nivara wild rice (NW1 and NW2) and the F1 offspring (NW-H). Although the Shannon index data in Asian cultivated rice (JaC and InC) were higher than they were in common wild rice (CW1 and CW2) and their F1 offspring (CW-H), no significant differences were observed in the Simpson, Chao1 and ACE indices of bacterial communities between Asian cultivated rice (JaC and InC), common wild rice (CW1 and CW2), and their F1 offspring (CW-H) (Fig. 2).

**Fig. 3:**
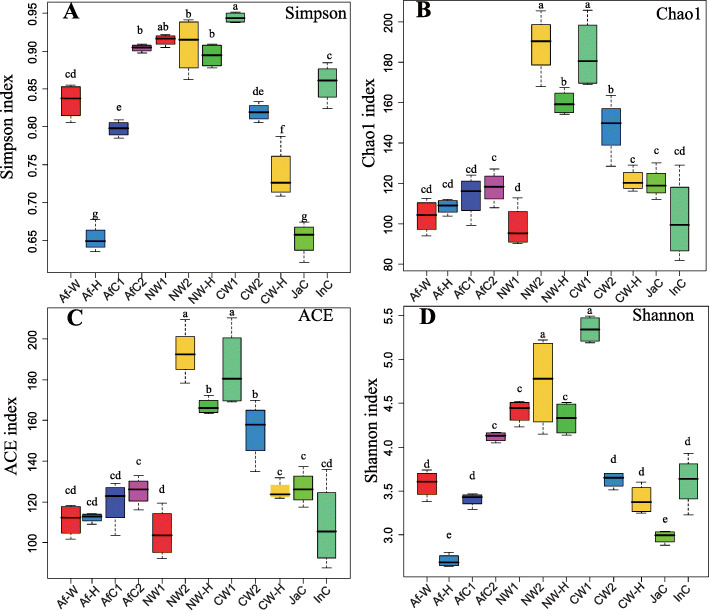
Box plots of the alpha diversity indices of the endophytic fungal communities of cultivated and wild rice plants, and their F1 offspring. Simpson (**a**), Chao1 (**b**), ACE (**c**) and Shannon (**d**) indices. Each boxplot represents the distribution of diversity present in four replicates (*n* = 4). Af-W, African wild rice; Af-H, F1 generation of a cross between African wild rice (Af-W) and African cultivated rice (AfC1); AfC1, African cultivated rice No. 2; AfC2, African cultivated rice No. 4; NW1, nivara wild rice No. 1; NW2, nivara wild rice No. 2; NW-H, F1 generation of a cross between nivara wild rice (NW1) and Asian cultivated rice (indica, InC); CW1, common wild rice No. 1; CW2, common wild rice No. 2; CW-H, F1 generation of a cross between common wild rice (CW1) and Asian cultivated rice (japonica, JaC); InC, Asian cultivated rice (Jiangxi indica); JaC, Asian cultivated rice (Jiangxi japonica)

The results of the alpha diversity analysis of the fungal communities indicated that there were no significant differences in the Simpson, Chao1, ACE and Shannon indices among African cultivated rice (AfC1 and AfC2), African wild rice (Af-W) and their F1 offspring (Af-H) (Fig. 3a-d). The Simpson and Shannon indices in the Asian rice group were significantly higher in nivara wild rice (NW1 and NW2) and F1 offspring (NW-H) than they were in cultivated rice (JaC and InC) (Fig. 3a, d), while the Chao1 and ACE indices were notably higher in wild rice (CW1 and CW2) than in cultivated rice (JaC and InC) and F1 offspring (CW-H) (Fig. 3b, c).

### Beta diversity analyses of the endophytic bacterial and fungal communities of roots in cultivated and wild rice species and their F1 offspring

**Fig. 4:**
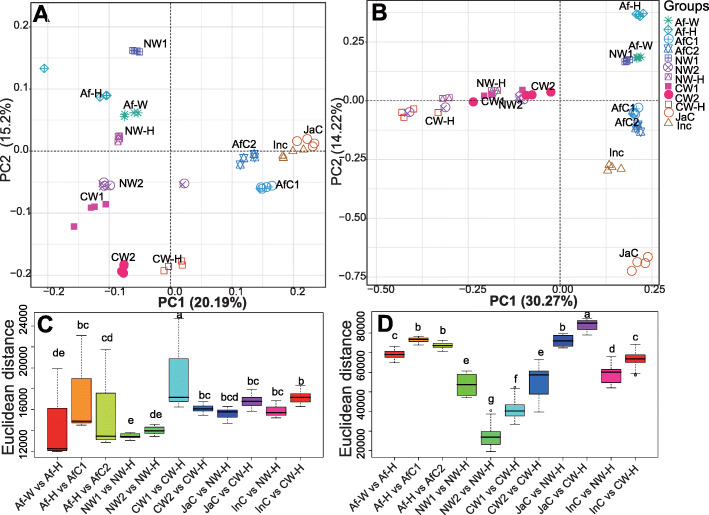
Principal component analysis of the bacterial (**a**) and fungal (**b**) communities, and Euclidean distances of bacterial (**c**) and fungal (**d**) communities based on the comparison of the F1 offspring with their wild and cultivated parents. Each boxplot represents the distribution of diversity present in four replicates (*n* = 4). Af-W, African wild rice; Af-H, F1 generation of a cross between African wild rice (Af-W) and African cultivated rice (AfC1); AfC1, African cultivated rice No. 2; AfC2, African cultivated rice No. 4; NW1, nivara wild rice No. 1; NW2, nivara wild rice No. 2; NW-H, F1 generation of a cross between nivara wild rice (NW1) and Asian cultivated rice (indica, InC); CW1, common wild rice No. 1; CW2, common wild rice No. 2; CW-H, F1 generation of a cross between common wild rice (CW1) and Asian cultivated rice (japonica, JaC); InC, Asian cultivated rice (Jiangxi indica); JaC, Asian cultivated rice (Jiangxi japonica)

A principal component analysis (PCA), based on Euclidean distances, was conducted separately for the endophytic bacterial and fungal communities of cultivated and wild rice species (Fig. 4). Results of the analysis of the bacterial communities revealed that Asian cultivated rice (JaC and InC) and African cultivated rice (AfC1 and AfC2) species clustered together, while indica and japonica accessions grouped together (Fig. 4a). African wild rice (Af-W) clustered together with the F1 offspring derived from a cross between African wild rice and African cultivated rice. Similarly, nivara and common wild rice species were more similar to their respective F1 offspring (Fig. 4a) than they were to Asian cultivated rice.

The PCA analysis of the compositions of the fungal communities revealed that the Asian (InC) and African (AfC1 and AfC2) species of cultivated rice clustered near each other, while the two accessions of African cultivated rice clustered very close to each other (Fig. 4b). NW2, NW-H, CW1, CW2 and CW-H grouped into cluster II, revealing a similarity in community structure between Asian wild rice (NW2, CW1 and CW2) and their F1 offspring (NW-H, CW-H) obtained from the crosses with Asian cultivated rice species (Fig. 4b). Similarly, the structures of the fungal communities of African wild rice (Af-W) and its F1 offspring (Af-H) were more similar than their African cultivated rice parents (AfC1 and AfC2) (Fig. 4b).

The Euclidean distances were then calculated to test if the distances between the different comparisons of wild and cultivated rice species were statistically significant (Fig. 4c, d). Results indicated that the differences in Euclidean distances obtained from the comparison of the bacterial communities between African wild rice and F1 offspring were not significant (Fig. 4c). Similarly, the differences in Euclidean distances in the comparison of the bacterial communities of nivara wild rice/common wild rice and F1 offspring were not statistically significant either. Our data also revealed that the Euclidean distances derived from the comparison of the fungal community compositions between African wild rice and F1 offspring (Af-W vs Af-H) were lower than the distances between cultivated rice and F1 offspring (Af-H vs AfC1 and Af-H vs AfC2) (Fig. 4d). Similarly, the Euclidean distances obtained for the comparison of fungal community compositions between cultivated rice and F1 offspring (JaC vs CW-H and InC vs CW-H) were higher than the distances between common wild rice and F1 offspring (CW1 vs CW-H and CW2 vs CW-H) (Fig. 4d). Furthermore, the Euclidean distances in the comparison of the fungal community structure between cultivated rice and F1 offspring (JaC vs NW-H and InC vs NW-H) were greater than the distances between common wild rice and F1 offspring (NW1 vs NW-H and NW2 vs NW-H) (Fig. 4d).

### Network analysis of bacterial and fungal OTUs

**Fig. 5:**
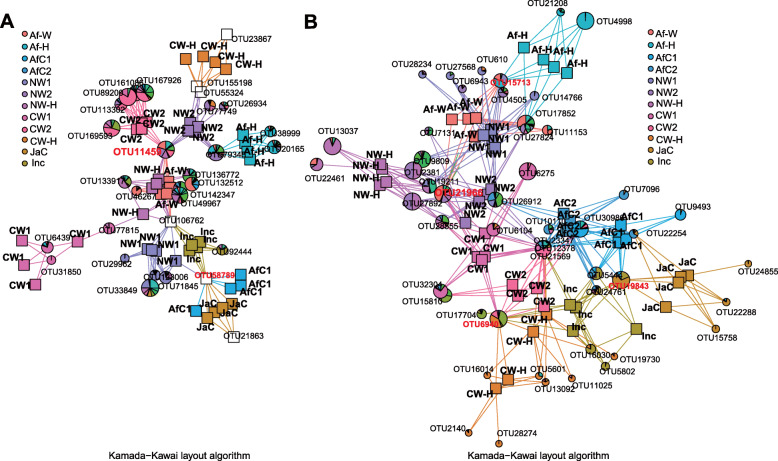
Co-occurrence network of bacterial (**a**) and fungal (**b**) communities in the endophytic microbiomes of cultivated and wild rice plants and their F1 offspring. The dot size corresponds to operational taxonomic unit (OTU) abundance (log_2_-transformed). Co-occurrence may be exaggerated among the large numbers of rare OTUs due to the dominance of zeros for rare OTUs in most samples. Each boxplot represents the distribution of diversity present in four replicates (*n* = 4). Af-W, African wild rice; Af-H, F1 generation of a cross between African wild rice (Af-W) and African cultivated rice (AfC1); AfC1, African cultivated rice No. 2; AfC2, African cultivated rice No. 4; NW1, nivara wild rice No. 1; NW2, nivara wild rice No. 2; NW-H, F1 generation of a cross between nivara wild rice (NW1) and Asian cultivated rice (indica, InC); CW1, common wild rice No. 1; CW2, common wild rice No. 2; CW-H, F1 generation of a cross between common wild rice (CW1) and Asian cultivated rice (japonica, JaC); InC, Asian cultivated rice (Jiangxi indica); JaC, Asian cultivated rice (Jiangxi japonica)

A Kamada-Kawai plot based on the relative OTU abundances in the samples was constructed to reveal the interaction mode of co-occurring community members, and to infer possible cooperation between different microbial groups [36]. Based on distribution of the relative abundance of the obtained OTUs for each taxon in different samples, we identified microbial groups that co-occurred with each other, and then constructed the co-occurrence network of dominant microbial groups to explore their ecological significance. The network analysis revealed that Asian and African cultivated rice accessions (Af-H, AfC2, JaC and InC) clustered together and had more significant correlations in both bacteria and fungi than what was observed for wild rice species (Fig. 5a, b). African wild rice (Af-W), common wild rice (CW2) and nivara wild rice (NW2) had one common bacterial species that was significantly correlated, namely OTU11459 (*Acidovorax* sp.) (Fig. 5b), while African wild rice (Af-W) and nivara wild rice (NW1 and NW2) had several common fungal species that were significantly correlated, namely OTU21966 (order Pleosporales), OTU15713 (genus *Myrothecium*) and OTU6940 (genus *Bullera*) (Fig. 5b). Furthermore, there were more bacterial and fungal taxa that were significantly correlated between both African and Asian wild rice and their F1 offspring than between cultivated rice and their F1 offspring (Fig. 5a, b). The core bacterial OTU that connected Asian and African wild rice with their F1 offspring was OTU11459 (e.g. *Acidovorax* sp.), while the core bacterial OTU that linked Asian and African cultivated rice was OTU58789 belongs to the genus *Bradyrhizobium* (Fig. 5a). Additionally, the core fungal OTUs that connected Asian wild rice with African wild rice, and with their F1 offspring were OTU21966 (Pleosporales), OTU15713 (*Myrothecium* sp.) and OTU6940 (*Bullera* sp.). The core fungal OTU that linked Asian and African cultivated rice was OTU19843, which belongs to the genus *Dendroclathra* (Fig. 5b).

## Discussion

The current study provided evidence of vertical transmission of specific endophytic microorganisms (fungi and bacteria) in plants. The propagation of fungi and bacteria in the offspring is different from their parents because the compositions of fungi and bacteria may change during the transmission [33]. Microbial diversity within a given community has been generally assessed using the total number of OTUs (richness), their relative abundance (Shannon diversity), or indices that combine these two parameters (evenness) [29, 37]. Studies have generally used microbial alpha diversity to evaluate the relationship between the structure and function of a microbial community within an ecosystem [38, 39]. Results of our study indicate that the alpha diversity and abundance indices of the bacterial and fungal endophytic root microbiomes of cultivated and wild rice species, as well as F1 offspring resulted from the crosses between wild and cultivated rice species, were not static and set but were rather dynamic. Significantly higher Chao1 and Shannon indices were obtained for the bacterial community compositions of both African and Asian cultivated rice, relative to their related wild rice and respective F1 offspring (Fig. 2b, d), indicating that bacterial communities in cultivated rice accessions were composed of a greater number of bacterial species and greater evenness than wild rice accessions. No stabilized significant differences were observed in the Simpson, Chao1, ACE and Shannon indices between African cultivated rice species and African wild rice species and their F1 offspring (Fig. 2a, b, c, d). The Simpson and Shannon indices of the fungal communities were significantly higher in nivara wild rice and F1 offspring than they were in cultivated rice and F1 offspring (Fig. 3a, d), indicating that nivara wild rice and its derived F1 offspring retained greater fungal diversity and evenness than Asian cultivated rice. The Chao1 and ACE indices in common wild rice were also higher than in cultivated rice and F1 offspring (Fig. 3b, c). Collectively, these findings indicate that there were a greater number of fungal species in common wild rice than there were in cultivated rice species. In support of this finding, Tian et al. (2017) demonstrated that the diversity and abundance indices of root-associated bacteria in *O. rufipogon* were higher than in *O. sativa* [29].

Beta diversity analyses have been widely used for the analysis of biological diversity in the composition of microbial communities along environmental gradients [40, 41]. In our study, the examined Asian and African cultivated rice species clustered together based on similarities in bacterial and fungal community structures, and the indica and japonica species grouped together, also based on similarity in microbial community composition and diversity (Fig. 4a, b). The African wild rice accessions, however, clustered together with the F1 offspring resulted from the crosses between African wild rice and African cultivated rice (Fig. 4a, b). Similarly, the bacterial and fungal community compositions of nivara and common wild rice species were more similar to their respective F1 offspring than they were to Asian cultivated rice (Fig. 4a, b). This demonstration of microbial transmission supports the idea that microbial consortia and their host constitute a combined unit of selection.

The community structure of African wild rice (Af-W) was more similar to its F1 offspring than it was to African cultivated rice. The compositions of the bacterial communities of the respective F1 offspring resulted from a cross between wild nivara and common wild rice with cultivated indica and japonica rice species were also more similar to those of nivara and common wild rice than they were to those of the indica and japonica species of cultivated rice (Fig. 4a). The fungal communities of African cultivated rice (AfC1 and AfC2) accessions were grouped very closely together, and they also clustered near to that of the Asian cultivated rice (InC) (Fig. 4b). The structures of the fungal communities in nivara wild rice and common wild rice and their respective F1 offspring were more similar to each other than they were observed in the fungal community structures of Asian cultivated rice accessions and their F1 offspring (Fig. 4b). Results also indicated that fungal community structures in African wild rice and its F1 offspring were more similar to each other than they were between African cultivated rice species and their F1 offspring (Fig. 4b). Furthermore, the Euclidean distances in the comparison of the fungal communities of African wild rice and the F1 offspring (Af-W vs Af-H) was smaller than it was between African cultivated rice (AfC1 and AfC2) and the F1 offspring (Fig. 4d), indicating that the F1 offspring were more similar to African wild rice than they were to African cultivated rice in regard to fungal community composition. Similarly, the Euclidean distances of the fungal communities in the comparison between Asian cultivated rice (JaC and InC) and F1 offspring were larger than those that were observed between common wild rice and F1 offspring, demonstrating that the F1 offspring exhibited a closer relationship to common and nivara wild rice species than they did to cultivated indica and japonica species of rice in regard to fungal community composition (Fig. 4d). Wild rice species and F1 offspring require less energy to produce seeds in their life cycle than cultivated rice, and wild rice and F1 offspring are perennial, while cultivated rice species are annual [42]. These similar features of the F1 offspring and the wild rice parents may partially explain why the F1 generation received a greater number of root endophytic fungi from the wild rice than from cultivated rice parents.

Network analysis of microbiomes has been used to explore co-occurrence patterns among microbial taxa and potential functions [43]. The transmission of a microbe along plant clonal networks extends the concept of physiological integration of information and resources previously demonstrated for microorganisms [44]. An integrated network of blueprint supports the concept of a meta-holobiont relationship in which plants act as sinks or sources of microorganisms [44]. Such a structure can ensure communication between plants, especially between parents and offspring, which contributes to the adaptability and/or fitness of clones as a whole [44]. In the present study, the conducted network analysis revealed that accessions of Asian and African cultivated rice species clustered together and had a higher number of significant correlations than wild rice for both bacteria and fungi (Fig. 5a, b). The correlated bacterial and fungal species that were common to African wild rice and common wild rice are presented in Fig. 5b. The bacterial communities associated with the rhizospheres of wild rice species have been shown to exhibit distinct differences in the communities associated with cultivated rice species (Fig. 5a), suggesting that root traits selected during the process of domestication have a significant influence on the composition on bacterial taxa in the rhizosphere of rice [45], which is supported by the study of Piromyou et al. [46]. Furthermore, Zhang et al. (2019) showed that rice genotype had little impact on the diversity and richness of endophytic bacteria in seeds [10], and Shi et al. (2018) reported that Glomeromycota had higher abundant in wild rice rhizosphere and could help wild rice in resisting *Magnaporthe oryzae* [28]. The network analysis revealed significantly different correlations of bacterial and fungal species with cultivated rice when compared with wild rice and their F1 offspring (Fig. 5a, b). *Acidovorax* was the core bacterial genus that was common to both Asian and African wild rice and their F1 offspring, while *Bradyrhizobium* was the core bacterial genus that was common to both Asian and African cultivated rice (Fig. 5a). *Acidovorax* is a pathogen of watermelon (*Citrullus vulgaris*), but some species may also play a role in stimulating plant immune systems [47, 48]. *Bradyrhizobium* improves nitrogen availability to plants under both normal and adverse environmental stress conditions [49–53]. *Bradyrhizobium,* as an endophyte, was demonstrated to improve rice growth, perhaps due to the ability of this bacterium to produce indole-3-acetic acid, 1-amino-cyclopropane-1-carboxylic acid deaminase, as well as its role in nitrogen fixation [53, 54]. Pongdet et al. also demonstrated that rice cultivars can regulate their association with endophytic bradyrhizobia [46]. Endophytic *Bradyrhizobium* in cultivated rice may assist plants in absorbing or utilizing nitrogen that can be used for plant growth-related processes, such as in photosynthesis in leaves [53, 55]. This study showed that cultivated rice accessions had higher relative abundance of *Bradyrhizobium* than wild rice accessions did (Fig. 5a), which may help cultivated rice in absorbing nitrogen. Pleosporales, *Myrothecium* and *Bullera* were the core fungal taxa that were common to wild rice and their F1 offspring, while *Dendroclathra* was the fungal genus that was common to cultivated rice and their F1 offspring (Fig. 5b). *Bullera* spp. have been known to function as biocontrol agents that improve disease resistance and growth in plants [56]. Therefore, the higher relative abundance of *Bullera* spp. in wild rice and their F1 offspring may have special relevance, contributing to disease resistance in wild rice by stimulating basal resistance.

## Conclusions

In the present study, we conducted a comprehensive comparison of the structures of the endophytic microbial communities in roots of Asian and African wild and cultivated rice species and their F1 offspring. The F1 generation obtained a greater number of root endophytic fungi from both Asian and African wild rice than from cultivated rice parents, which shows the significant impact of rice hybridization on the establishment of the endophytic microbiota in the offspring and may provide ideas for development of useful biofertilizers in future. Furthermore, higher relative abundances of the fungal order Pleosporales, and the genera *Myrothecium* and *Bullera* were found in African and Asian wild rice and F1 offspring, while the fungal genus *Dendroclathra* exhibited its highest relative abundances in African and Asian cultivated rice species based on the results obtained from the network analysis. The core bacterial genus that was common to African and Asian wild rice and their F1 offspring was *Acidovorax*, while the core bacterial genus that was common to African and Asian cultivated rice was *Bradyrhizobium*.

## Methods

### Plant materials

The Asian and African accessions of wild and cultivated rice species along with the F1 generation accessions used in this study are listed in Table 1. Seeds of the wild rice species *O. barthii*, *O. nivara* and *O. ruffipogon* were kindly provided by the International Rice Research Institute (IRRI), and seeds of Asian cultivated rice (*O. sativa *subsp. *indica* and *O. sativa *subsp. *japonica*) and African cultivated rice (*O. glaberrima*) were obtained from the Jiangxi Academy of Agricultural Sciences (Table 1). The obtained seeds were planted in Hainan Province, China. Seeds of the F1 generation of crosses between (i) African wild rice (Af-W) × African cultivated rice No. 2 (AfC1), (ii) nivara wild rice No. 1 (NW1) × Asian cultivated rice indica (InC), and (iii) common wild rice No. 1 (CW1) × Asian cultivated rice japonica (JaC) were also grown in Hainan Province. The wild and cultivated rice varieties, as well as their F1 offspring, were all AA genotypes.

**Table 1:** Wild and cultivated rice accessions, and their F1 offspring used in this study. IRRI, International Rice Research Institute

Species	Seed source	Sample name	Genome	Distribution
*Oryza barthii* (African wild rice)	IRRI	Af-W	AA	Western, eastern and southern Africa
*O. glaberrima* (African cultivated rice No. 2)	Jiangxi, China	AfC1	AA	West Africa
*O. glaberrima* (African cultivated rice No. 4)	Jiangxi, China	AfC2	AA	
African wild rice × African cultivated rice No. 2	IRRI	Af-H	AA	Western, eastern and southern Africa
*O. nivara* (nivara wild rice No. 1)	IRRI	NW1	AA	Tropical and sub-tropical Asia
*O. nivara* (nivara wild rice No. 2)	IRRI	NW2	AA	
nivara wild rice No. 1 × Asian cultivated rice indica	IRRI	NW-H	AA	
*O. ruffipogon* (common wild rice No. 1)	IRRI	CW1	AA	Tropical and sub-tropical Asia
*O. ruffipogon* (common wild rice No. 2)	IRRI	CW2	AA	
common wild rice No. 1 × Asian cultivated rice japonica	IRRI	CW-H	AA	
*O. sativa* subsp. *indica* (Asian cultivated rice, indica)	Jiangxi, China	InC	AA	China
*O. sativa* subsp. *japonica* (Asian cultivated rice, japonica)	Jiangxi, China	JaC	AA	

Seeds were surface-sterilized by soaking them in a 2% sodium hypochlorite solution for 5 min, and subsequently rinsing twice with sterilized water. The sterilized seeds were placed in 9-cm petri dishes containing 2 mL water to induce germination. The germinated seeds were transplanted into pots containing 3 Kg soil/pot (18-cm high and 20-cm diameter at the top) with 4 seeds in each pot. Each designated group of accessions comprised 4 replicates. The pots were placed in a growth chamber set at 26/20 °C and a relative humidity of 65%, under a 16-h light/8-h dark photoperiod. The pots were watered once every 3 days with the same volume of Hoagland’s nutrient solution to maintain a relative soil water content of 13–15%. Whole plants were collected at 40 days after transplanting. Roots were cut and cleaned with tap water and sterilized by soaking them in 1% sodium hypochlorite for 5 min, followed by rinsing them three times with 50 mL sterilized water.

### DNA extraction and high-throughput DNA sequencing

Sterilized roots from plants of each of the accessions were frozen in liquid nitrogen and subsequently ground into powder. DNA was subsequently extracted from the ground root powder using a Fast DNA SPIN Kit (Catalog No. 6560–220, Germany) according to the manufacturer’s instructions. A total of 0.5 g of root powder from each sample was used for each extraction. The extracted DNA was quantified using a NanoDrop 2000 (Thermo Scientific, Germany) prior to PCR amplification of the V3-V4 hypervariable regions of 16S rRNA using the primer set 341F 5′-ACTCCTACGGGAGGCAGCA-3′ and 785R 5′-GGACTACHVGGGTWTCTAAT-3′. The amplicons were used for the analysis of bacterial taxa. The ITS1 region was amplified using the primer set ITSF 5′-CTTGGTCATTTAGAGGAAGTAA-3′ and ITSR 5′-GCTGCGTTCTTCATCGATGC-3′. The resulting amplicons were used for the analysis of fungal taxa. The 2 × 250 bp paired-end sequences of the PCR amplicons were sequenced on a HiSeq platform (Illumina, San Diego, CA, USA) at the Beijing Biomarker Corporation (Beijing, China).

QIIME software (http://qiime.org/) was used to remove the barcodes and primers of the raw sequencing data and remove low-quality and other disqualified reads. The clean data were subjected to an RDP classifier for taxonomic assignment with a minimum of 50 confidence estimates. Random resampling was performed using the smallest sequences of all the samples. Based on a 97% similarity level, the OTUs were classified using USEARCH (http://www.drive5.com/usearch/Usearch) after removing singleton reads. A detrended correspondence analysis (DCA) was used to investigate the changes in the overall microbial community composition. A partial Mantel test was performed to correlate the microbial communities with factors based on Bray-Curtis distances.

### Data analyses

Alpha diversity indices (Simpson, Chao1, ACE, and Shannon) of the bacterial and fungal communities in the different accessions were calculated in QIIME based on rarefied samples. Significant differences in the diversity indices across samples were determined using a one-way ANOVA followed by a least significant difference (LSD) test with the aid of SPSS 19.0 software. PCA, based on OTU relative abundance, was performed for beta diversity assessment using the PCA function in the FactoMineR package of R software version 3.2.1 [57]. Euclidean distances were calculated and used in the PCA analysis. The network analysis to determine the correlations among the taxa in bacterial and fungal communities was performed using the dominant OTUs in each sample [36].

## Supplementary Information

**Additional file 1: Table S1.** Summary of the statistics of the high-throughput sequencing data for bacteria and fungi. Each group comprised 4 replicates (*n* = 4) indicated by A, B, C and D. Af-W, African wild rice; Af-H, F1 generation of a cross between African wild rice (Af-W) and African cultivated rice (AfC1); AfC1, African cultivated rice No. 2; AfC2, African cultivated rice No. 4; NW1, nivara wild rice No. 1; NW2, nivara wild rice No. 2; NW-H, F1 generation of a cross between nivara wild rice (NW1) and Asian cultivated rice (indica, InC); CW1, common wild rice No. 1; CW2, common wild rice No. 2; CW-H, F1 generation of a cross between common wild rice (CW1) and Asian cultivated rice (japonica, JaC); InC, Asian cultivated rice (Jiangxi indica); JaC, Asian cultivated rice (Jiangxi japonica).

**Additional file 2: Figure S1.** Rarefication curves for the bacterial (**A**) and fungal (**B**) OTUs in each group. Each group was comprised of 4 replicates (*n* = 4). Af-W, African wild rice; Af-H, F1 generation of a cross between African wild rice (Af-W) and African cultivated rice (AfC1); AfC1, African cultivated rice No. 2; AfC2, African cultivated rice No. 4; NW1, nivara wild rice No. 1; NW2, nivara wild rice No. 2; NW-H, F1 generation of a cross between nivara wild rice (NW1) and Asian cultivated rice (indica, InC); CW1, common wild rice No. 1; CW2, common wild rice No. 2; CW-H, F1 generation of a cross between common wild rice (CW1) and Asian cultivated rice (japonica, JaC); InC, Asian cultivated rice (Jiangxi indica); JaC, Asian cultivated rice (Jiangxi japonica).

## Data Availability

All raw sequencing data in this article are available at the National Center for Biotechnology Information through the link https://www.ncbi.nlm.nih.gov/bioproject/PRJNA667520, bioproject number: PRJNA667520, SRA accession: SRP289931.

## Abbreviations

OTUs: Operational taxonomic units
PCA: Principal component analysis
IRRI: International Rice Research Institute
DCA: Detrended correspondence analysis
LSD: Least significant difference
