# Supplementary material for: Wild rice harbors more root endophytic fungi than cultivated rice in the F1 offspring after crossbreeding
Source: BMC Genomics. 2021 Apr 17;22:278. doi: 10.1186/s12864-021-07587-1 (PMC8052703; doi:10.1186/s12864-021-07587-1)
Supplement: Supplementary file 1 — Additional file 1: Table S1. Summary of the statistics of the high-throughput sequencing data for bacteria and fungi. Each group comprised 4 replicates (n = 4) indicated by A, B, C and D. Af-W, African wild rice; Af-H, F1 generation of a cross between African wild rice (Af-W) and African cultivated rice (AfC1); AfC1, African cultivated rice No. 2; AfC2, African cultivated rice No. 4; NW1, nivara wild rice No. 1; NW2, nivara wild rice No. 2; NW-H, F1 generation of a cross between nivara wild rice (NW1) and Asian cultivated rice (indica, InC); CW1, common wild rice No. 1; CW2, common wild rice No. 2; CW-H, F1 generation of a cross between common wild rice (CW1) and Asian cultivated rice (japonica, JaC); InC, Asian cultivated rice (Jiangxi indica); JaC, Asian cultivated rice (Jiangxi japonica). [file 12864_2021_7587_MOESM1_ESM.docx]

**Table S1**. Summary of the statistics of the high-throughput sequencing data for bacteria and fungi. Each group comprised 4 replicates (*n* = 4) indicated by A, B, C and D. Af-W, African wild rice; Af-H, F1 generation of a cross between African wild rice (Af-W) and African cultivated rice (AfC1); AfC1, African cultivated rice No. 2; AfC2, African cultivated rice No. 4; NW1, nivara wild rice No. 1; NW2, nivara wild rice No. 2; NW-H, F1 generation of a cross between nivara wild rice (NW1) and Asian cultivated rice (indica, InC); CW1, common wild rice No. 1; CW2, common wild rice No. 2; CW-H, F1 generation of a cross between common wild rice (CW1) and Asian cultivated rice (japonica, JaC); InC, Asian cultivated rice (Jiangxi indica); JaC, Asian cultivated rice (Jiangxi japonica).

| **Bacteria** | | | | **Fungi** | | | |
| --- | --- | --- | --- | --- | --- | --- | --- |
| **Sample** | **Total pairs** | **Clean reads** | **Percentage**  **(%)** | **Sample** | **Total pairs** | **Clean reads** | **Percentage**  **(%)** |
| Af-WA | 52,731 | 46,726 | 88.61% | Af-WA | 45,278 | 41,962 | 93% |
| Af-WB | 51,200 | 45,442 | 88.75% | Af-WB | 46,339 | 40,708 | 88% |
| Af-WC | 51,638 | 46,017 | 89.11% | Af-WC | 51,311 | 47,136 | 92% |
| Af-WD | 51,422 | 46,075 | 89.60% | Af-WD | 48,732 | 43,829 | 90% |
| Af-HA | 48,744 | 43,325 | 88.88% | Af-HA | 43,840 | 41,747 | 95% |
| Af-HB | 42,795 | 38,118 | 89.07% | Af-HB | 49,402 | 47,138 | 95% |
| Af-HC | 34,863 | 30,750 | 88.20% | Af-HC | 50,429 | 47,860 | 95% |
| Af-HD | 46,102 | 40,785 | 88.47% | Af-HD | 49,591 | 47,306 | 95% |
| AfC1A | 50,073 | 44,468 | 88.81% | AfC1A | 39,198 | 32,642 | 83% |
| AfC1B | 46,394 | 40,906 | 88.17% | AfC1B | 48,572 | 41,611 | 86% |
| AfC1C | 51,310 | 45,537 | 88.75% | AfC1C | 49,798 | 42,647 | 86% |
| AfC1D | 55,390 | 48,816 | 88.13% | AfC1D | 48,673 | 40,916 | 84% |
| AfC2A | 40,779 | 36,304 | 89.03% | AfC2A | 49,319 | 41,286 | 84% |
| AfC2B | 44,149 | 39,381 | 89.20% | AfC2B | 50,836 | 42,989 | 85% |
| AfC2C | 47,805 | 42,279 | 88.44% | AfC2C | 49,433 | 42,390 | 86% |
| AfC2D | 52,858 | 46,893 | 88.72% | AfC2D | 51,031 | 41,680 | 82% |
| NW1A | 49,884 | 44,381 | 88.97% | NW1A | 48,541 | 46,036 | 95% |
| NW1B | 41,744 | 37,257 | 89.25% | NW1B | 42,781 | 40,692 | 95% |
| NW1C | 47,183 | 41,960 | 88.93% | NW1C | 44,110 | 41,622 | 94% |
| NW1D | 50,989 | 45,429 | 89.10% | NW1D | 46,838 | 44,600 | 95% |
| NW2A | 48,704 | 43,547 | 89.41% | NW2A | 46,668 | 39,317 | 84% |
| NW2B | 49,823 | 44,427 | 89.17% | NW2B | 40,851 | 34,138 | 84% |
| NW2C | 37,758 | 34,327 | 90.91% | NW2C | 50,483 | 43,243 | 86% |
| NW2D | 36,134 | 32,516 | 89.99% | NW2D | 36,273 | 30,748 | 85% |
| NW-HA | 37,754 | 34,009 | 90.08% | NW-HA | 40,581 | 36,362 | 90% |
| NW-HB | 35,201 | 31,633 | 89.86% | NW-HB | 45,669 | 40,100 | 88% |
| NW-HC | 41,072 | 36,950 | 89.96% | NW-HC | 38,755 | 34,428 | 89% |
| NW-HD | 36,059 | 32,589 | 90.38% | NW-HD | 43,057 | 37,828 | 88% |
| CW1A | 43,784 | 39,861 | 91.04% | CW1A | 39,845 | 36,756 | 92% |
| CW1B | 39,011 | 35,605 | 91.27% | CW1B | 35,588 | 33,194 | 93% |
| CW1C | 38,898 | 35,401 | 91.01% | CW1C | 36,923 | 34,301 | 93% |
| CW1D | 33,883 | 30,814 | 90.94% | CW1D | 39,854 | 36,924 | 93% |
| CW2A | 41,540 | 37,728 | 90.82% | CW2A | 40,206 | 36,954 | 92% |
| CW2B | 41,790 | 38,169 | 91.34% | CW2B | 39,020 | 35,971 | 92% |
| CW2C | 42,793 | 38,712 | 90.46% | CW2C | 42,386 | 39,035 | 92% |
| CW2D | 41,446 | 37,537 | 90.57% | CW2D | 38,924 | 35,904 | 92% |
| CW-HA | 42,629 | 37,912 | 88.93% | CW-HA | 41,422 | 37,808 | 91% |
| CW-HB | 36,486 | 32,531 | 89.16% | CW-HB | 43,540 | 39,732 | 91% |
| CW-HC | 39,269 | 35,719 | 90.96% | CW-HC | 38,791 | 35,004 | 90% |
| CW-HD | 36,528 | 32,818 | 89.84% | CW-HD | 41,373 | 37,298 | 90% |
| InCA | 64,401 | 57,034 | 88.56% | InCA | 42,625 | 39,450 | 93% |
| InCB | 37,453 | 33,656 | 89.86% | InCB | 41,280 | 38,356 | 93% |
| InCC | 36,805 | 32,678 | 88.79% | InCC | 43,975 | 40,680 | 93% |
| InCD | 60,778 | 54,230 | 89.23% | InCD | 42,432 | 39,485 | 93% |
| JaCA | 41,332 | 36,491 | 88.29% | JaCA | 46,158 | 38,465 | 83% |
| JaCB | 38,701 | 34,523 | 89.20% | JaCB | 40,185 | 33,343 | 83% |
| JaCC | 40,600 | 36,191 | 89.14% | JaCC | 46,587 | 37,807 | 81% |
| JaCD | 47,332 | 42,055 | 88.85% | JaCD | 55,597 | 45,902 | 83% |
| **Average** | **44,292** | **39,594** | **89%** | **Average** | **44,439** | **39,694** | **89%** |
| **Total** | **2,126,017** | **1,900,512** |  | **Total** | **2,133,100** | **1,905,330** |  |
