# Supplementary material for: Wild rice harbors more root endophytic fungi than cultivated rice in the F1 offspring after crossbreeding
Source: BMC Genomics. 2021 Apr 17;22:278. doi: 10.1186/s12864-021-07587-1 (PMC8052703; doi:10.1186/s12864-021-07587-1)
Supplement: Supplementary file 2 — Additional file 2: Figure S1. Rarefication curves for the bacterial (A) and fungal (B) OTUs in each group. Each group was comprised of 4 replicates (n = 4). Af-W, African wild rice; Af-H, F1 generation of a cross between African wild rice (Af-W) and African cultivated rice (AfC1); AfC1, African cultivated rice No. 2; AfC2, African cultivated rice No. 4; NW1, nivara wild rice No. 1; NW2, nivara wild rice No. 2; NW-H, F1 generation of a cross between nivara wild rice (NW1) and Asian cultivated rice (indica, InC); CW1, common wild rice No. 1; CW2, common wild rice No. 2; CW-H, F1 generation of a cross between common wild rice (CW1) and Asian cultivated rice (japonica, JaC); InC, Asian cultivated rice (Jiangxi indica); JaC, Asian cultivated rice (Jiangxi japonica). [file 12864_2021_7587_MOESM2_ESM.docx]

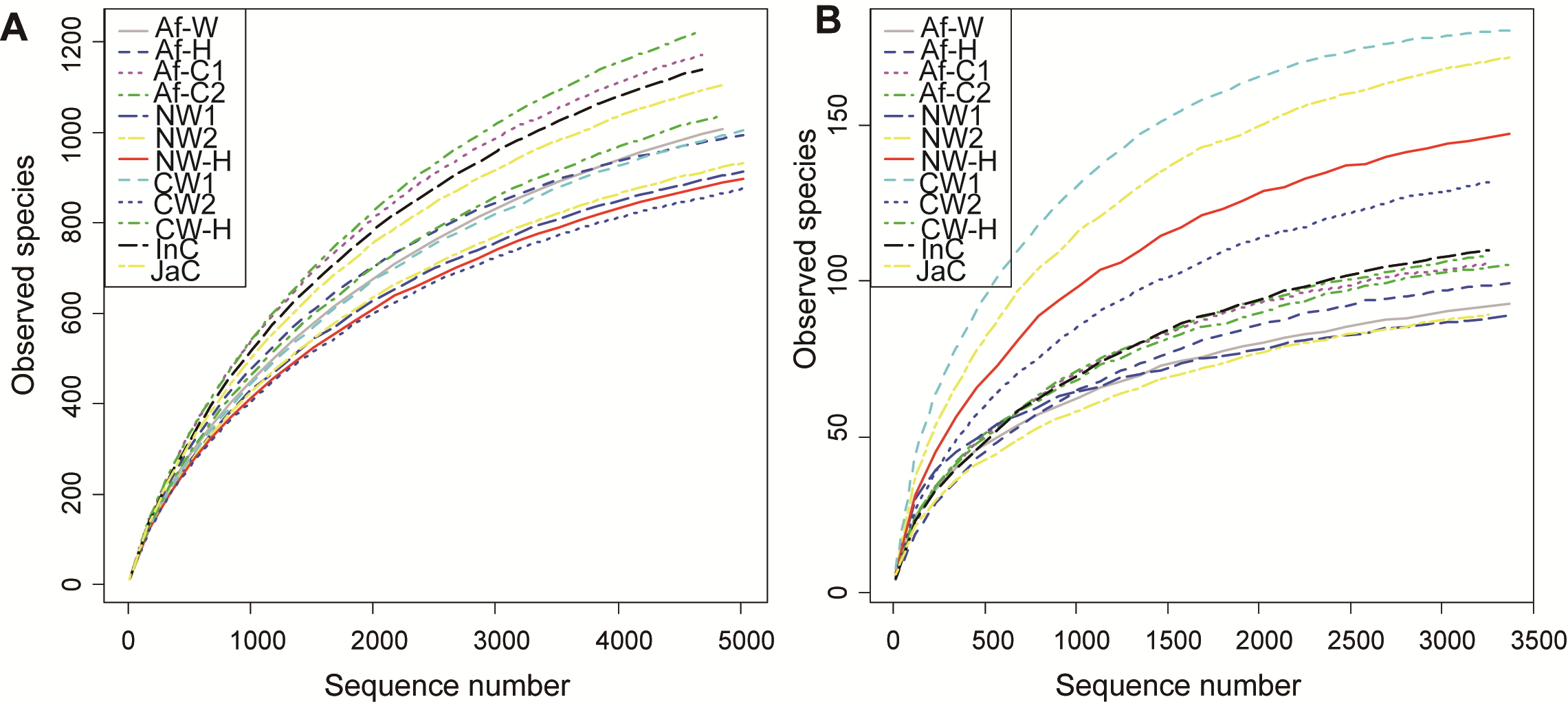


**Additional file 2: Figure S1** Rarefication curves for the bacterial (**A**) and fungal (**B**) OTUs in each group. Each group was comprised of 4 replicates (*n* = 4). Af-W, African wild rice; Af-H, F1 generation of a cross between African wild rice (Af-W) and African cultivated rice (AfC1); AfC1, African cultivated rice No. 2; AfC2, African cultivated rice No. 4; NW1, nivara wild rice No. 1; NW2, nivara wild rice No. 2; NW-H, F1 generation of a cross between nivara wild rice (NW1) and Asian cultivated rice (indica, InC); CW1, common wild rice No. 1; CW2, common wild rice No. 2; CW-H, F1 generation of a cross between common wild rice (CW1) and Asian cultivated rice (japonica, JaC); InC, Asian cultivated rice (Jiangxi indica); JaC, Asian cultivated rice (Jiangxi japonica).
